# Noninvasive nasopharyngeal proteomics of COVID-19 patient identify abnormalities related to complement and coagulation cascade and mucosal immune system

**DOI:** 10.1371/journal.pone.0274228

**Published:** 2022-09-12

**Authors:** Mohamad Ammar Ayass, Wanying Cao, Jin Zhang, Jun Dai, Kevin Zhu, Trivendra Tripathi, Natalya Griko, Victor Pashkov, Lina Abi-Mosleh

**Affiliations:** Ayass Bioscience LLC, Frisco, TX, United States of America; Waseda University: Waseda Daigaku, JAPAN

## Abstract

Serum or plasma have been the primary focus of proteomics studies for COVID-19 to identity biomarkers and potential drug targets. The nasal mucosal environment which consists of lipids, mucosal immune cells, and nasal proteome, has been largely neglected but later revealed to have critical role combating SARS-CoV-2 infection. We present a bottom-up proteomics investigation of the host response to SARS-CoV-2 infection in the nasopharyngeal environment, featuring a noninvasive approach using proteins in nasopharyngeal swabs collected from groups of 76 SARS-CoV-2 positive and 76 negative patients. Results showed that 31 significantly down-regulated and 6 up-regulated proteins were identified (*p* < 0.05, log_2_ FC > 1.3) in SARS-CoV-2 positive patient samples as compared to the negatives; these proteins carry potential value as markers for the early detection of COVID-19, disease monitoring, as well as be drug targets. The down-regulation of coagulation factor 5 indicates a thrombotic abnormality in COVID-19 patients and the decreased IgG4 suggests an abnormal immune response at the point of entry in human nasopharyngeal environment, which is in consistent with KEGG and GO pathway analysis. Our study also demonstrated that mass spectrometry proteomics analysis of nasopharyngeal swabs can be used as a powerful early approach to evaluate host response to SARS-CoV-2 viral infection.

## Introduction

Since December 2019, severe acute respiratory syndrome coronavirus 2 (SARS-CoV-2) has caused a global pandemic—an acute respiratory disease known as coronavirus disease 2019 (COVID-19). As of Jan 7th, 2022, 298,915,721 confirmed cases of COVID-19, including 5,469,303 deaths have been reported to WHO globally [1]. Although global wide vaccination has dampened the spread of SARS-CoV-2, the high speed of virus mutation is continuously creating a giant burden on public resources, leading to more hospitalizations, and potentially more deaths according to the Centers for Disease Control and Prevention [2]. Symptoms for COVID-19 patients varied from mild to critical; most (81%) had mild symptoms, 14% were severe, and 5% were critical [3]. Although the molecular mechanisms involved in the pathogenesis of SARS-CoV-2 infection are not fully understood, they have been and continue to be deeply investigated worldwide. Several risk factors for severe morbidity and/or mortality related to COVID-19 have been identified, including older age, obesity, diabetes, and high blood pressure [4].

Multi-omics techniques have been widely employed to investigate the biology of SARS-CoV-2 infection and biomarkers of potential drug targets [5–7]. By investigating the proteomics, metabolomics, transcriptomics, and secretomics of the peripheral blood mononuclear cells in COVID-19 patients, Stephenson et al. identified a major immunological shift between SARS-CoV-2 mild and moderate infection and suggested that the drop in blood nutrients, increased inflammation and the emergence of novel immune cell subpopulations intensify with disease severity [7]. Serum proteomics has been especially useful to identify more human immune changes and biomarkers of the pathogenesis of SARS-CoV-2 infections [8, 9]. Among all pathogenesis mechanisms, several clinical reports together with multiple serum proteomics studies suggested that complement activation is a key contributor to the COVID-19 pathogenesis, which has been thoroughly reviewed elsewhere [10].

Multiple studies have revealed the critically important role of systemic immune system in SARS-CoV-2 infections [11, 12]. The mucosal immunity, however, has been neglected for a long time, but tends to be a critical aspect of SARS-CoV-2 infection, especially in clearance of the virus [13–15]. Nasal mucus consists of a variety of lipids, glycoconjugates, immune cells, cellular debris, and proteins that protect the epithelium airway [16]. The antibodies from a network of immune cells that reside underneath the upper airway mucosal membranes, varieties of enzymes, enzyme inhibitors, antioxidants, antibacterial proteins, and proteins exudated from plasma, form a key front-line defense against infectious pathogens and particulate pollutants in the air [16–18]. Together with the microbial organisms that are ubiquitous in the environment, the nasopharyngeal microenvironment has evolved highly specialized functions to protect the lower respiratory tract, and has been shown to play a central role in the transmission, modulation, and clinical progression of SARS-CoV-2 infection [19]. Dysregulation of the critical homeostatic and defensive functions of the mucosal system allows pathogens to enter the lower respiratory tract, potentially leading to serious systemic symptoms [18].

Drugs and vaccines that could utilize the biomarkers in the nasopharyngeal enviroment and mucosal immune system to produce antibodies in the nasal area should help stop the coronavirus at the point of entry. Therefore, identification of biomarkers in the nasopharyngeal microenvironment associated with viral clearance, vaccine implementation, potential drug targets, and easily measurable correlates of protection can allow monitoring of infection-induced immunity and facilitate novel drug/vaccine development [17]. Our study proposes to use mass spectrometry-based proteomics to reflect the host response to SARS-CoV-2 viral infection in the nasopharyngeal environment and to identify new biomarkers that can be used for designing non-invasive prognostic tools and treatments at the point of entry of the virus.

## Materials and methods

### Materials

Reagents for Viral Transport Media (VTM) from Hank’s balanced salt solution (HBSS), fetal bovine serum, gentamicin, and Amphotericin B were obtained from Thermofisher Scientific (MA, USA). Reagents for MS sample preparation: acetone, methanol, urea, NaCl, MgCl_2_, Tris-HCl, and formic acid from Millipore sigma (MO, USA), and Pierce C18 centrifugal columns, HPLC-grade acetonitirle, and HPLC-grade water from Thermofisher Scientific.

### Subject recruitment and swab collection

This research was reviewed and approved by the Salus IRB Review Board (protocol number#ABS001_01_01_2022). The research was involved with no more than minimal risk and the data used for the research was de-identified. The full waiver of informed consent was approved by Salus IRB. The aim of the study is to develop a straightforward bottom-up mass spectrometry method to identify biomarkers in the nasopharyngeal microenvironment of SARS-CoV-2 positive patients by re-using the samples collected for RT-PCR test. All patient samples were collected from Ayass Laboratory in Frisco, Texas, and all patients consented to allow their samples be collected for research purpose. All nasopharyngeal swabs were collected by a medical practitioner practicing proper infection control. A sterile cotton swab was used to collect the nasopharyngeal specimen and stored in a 15 *m*l falcon tube containing 2 *m*l viral transport media (VTM) (Hank’s balanced salt solution (HBSS), 2% fetal bovine serum, 100 *μ*g/*m*l of gentamicin, and 0.5 *μ*g/*m*l of Amphotericin B) at room temperature. Samples were first tested by RT-PCR and classified according to CT values. 76 samples with a N1 CT value lower than 15 were used as SARS-CoV-2 positive samples and 76 samples with a CT value higher than 40 for both N1 and N2 were used as negative samples.

### SARS-CoV-2 RT PCR assay

Material:RNAdvance Viral Kit (Catalog Number A35604, Agencourt RNAdvance, Beckman Coulter) for automated viral RNA extraction on the Biomek I5 Laboratory Automation Workstation. 2019-nCoV RUO Kit, 500 rxn (cat# 10006713, IDT), 2019-nCoV_N_Positive Control (cat# 268335814 IDT); Hs_RPP30 Positive Control (cat# 268335815, IDT), TaqPathTM 1-Step RT-qPCR Master Mix (4x) (cat# A15299, TermoFisher scientific). Biomek i5 (cat# B87583, Beckman Coulter), StepOnePlus^*™*^ Real-Time PCR System (cat# 4376600, ThermoFisher Scientific). Biomek i5 (cat# B87583, Beckman Coulter), StepOnePlus^*™*^ Real-Time PCR System (cat# 4376600, ThermoFisher Scientific).

Viral RNA extraction from nasopharyngeal swabs. RNA was extracted from nasopharyngeal swabs collected in viral transport media using RNAdvance Viral Kit which is based on paramagnetic bead technology. Automated extraction was performed on the Biomek I5 systems using the standard protocol provided by Beckman Coulter for Viral Nucleic Acid Extraction from Swabs using RNAdvance Viral Kit.

SARS-CoV-2 RT PCR Assay. The purified nucleic acids are reverse transcribed into cDNA and amplified using the TAQPATH 1-STEP RT-QPCR MM and the Applied Biosystems^*™*^ StepOnePlus Real-Time PCR instrument using primers and probes for N1, N2, and RP detection from IDT. In the process, the probes anneal to two specific SARS-CoV-2 target sequences and one human target sequence located between two unique forward and reverse primers for the following genes: N protein sequence N1, N protein sequence N2 and human RNP protein. The procedure identifies two SARS-CoV-2 targets as well as a human target gene as an internal control. RT-PCR was performed as describe in package insert for TaqPath^*™*^ 1-Step RT-qPCR cat# A15299, briefly: for each primer/probe set, we prepared 20 *u*l reaction mixture using 8.5 *u*l of nuclease-free water, 1.5 *u*l of primer/ probe mixture, 5 *u*l of TaqPath^*™*^ 1-Step RT-qPCR Master mix and 5 *u*l of extracted RNA. For revers transcription and amplification, we used the followed program: 2 min at 25°C; 15 min at 50°C, 2 min at 95°C and 45 cycles (3 sec at 95°C and 30 sec for 55°C).

### Sample preparation for a bottom-up mass spectrometry

Protein extraction from nasopharyngeal swabs was based on a published protocol [20] and improved based on the specific mass spectrometry instrument on-site. Virus and proteins in 200 *μ*l of samples in VTM were heat denatured at 65℃ for 45 mins and then precipitated with 600 *μ*l of 50/50 (v/v) acetone/methanol in -20℃ overnight. Samples were then centrifuged at 15,000g for 20 min at 4℃, then precipitated proteins were reconstituted with 75 *μ*l of 8M Urea lysis buffer (8M Urea, 75*mM* NaCl, 1*mM* MgCl_2_, Tris-HCl 50 *mM*). Unless otherwise noted, chemicals were purchased from Millipore sigma (MO, USA). After ensuring pellets are completely dissolved, protein quantification was conducted using BCA assay (Thermofisher Scientific, MA). A total of 30 *μ*g of proteins in each sample was reduced in 30 *μ*l of Urea lysis buffer with 10 mM Dithiothreitol at 95℃ for 10 min and alkylated with 15 mM of Iodoacetamide at room temperature in dark for 30 min. Processed samples were diluted 8-fold with a trypsin dilution buffer (1mM CaCl_2_ in 25 mM Tris-HCl, *pH* = 8) and then digested with mass spectrometry grade trypsin (Promega, WI) (1:50 w/w, 37℃ overnight). Digested peptides were desalted using Pierce C18 centrifugal columns (Thermofisher Scientific, MA) according to the manufacturer’s instructions. The desalted peptides were dried in a centrifugal evaporator and reconstituteD with 30 *μ*l of 0.1% formic acid (FA) in H_2_O with 3% acetonitrile (ACN) for a bottom-up LC-MS/MS mass spectrometry analysis. Samples were processed in duplicate and injected in duplicate (*n* = 4 in total).

### LC-MS/MS analysis

For each sample, 1.5 *μ*l of reconstituted peptide mixture was separated on a Thermo Scientific UltiMateTM 3000 RSLCnano system using a Thermo Scientific PepMapTM RSLC C18 column (2*μ*m, 100 Å, 75 *μm* × 25 cm) at a flow rate of 300 nL/min. Peptides were separated using a linear gradient with 2–32% solvent B over 90 min and 32–95% solvent B for 15 min (solvent A: 0.1% FA in H2O, solvent B: 0.1% FA in ACN). A TOP 10 data-dependent acquisition method was conducted on a Thermo Scientific Q Exactive Mass Spectrometry with the following parameters: full scan: 70,000 resolution, 375–1,500 m/z scan range, 3E+6 automatic gain control target, 100 ms maximum injection times; MS2: 17,500 resolution, 1E+5 automatic gain control target, 60ms maximum injection times, 2.0 m/z isolation window, normalized collision energy of 27, minimum AGC target at 8E+3 for an intensity threshold at 1.3E+5.

### LC-MS/MS data processing

Proteome Discoverer 2.2 (PD) (Thermofisher Scientific, MA) was employed for a label-free quantification using SequestHT against human proteome (26,152,582 sequences) and SARS-CoV-2 virus database (14,230) downloaded from Uniprot (Mar 15th, 2021). Fixed modification included carbamidomethylation, and up to three dynamic modifications were allowed including methionine oxidation and asparagine/glutamine deamidation. The precursor mass tolerance was 10 ppm and the fragment mass tolerance was 0.02 Da. Trypsin cleavage (KR/not P) was set as enzyme cleavage with a maximum missed cleavage of 2. For precursor ion label-free quantification, a Minora Feature Detector was used in the processing workflow. Both unique and razor peptides were used for quantification and results were normalized based on total peptides amount. Data were analyzed using PD in 9 different batches, later incorporated in one .csv via keeping proteins that consistently identified across 80% of replicates, and further normalized using Normalizer to choose a proper normalization method [21, 22]. The pathway analysis was conducted using GO-based and KEGG-based enrichment analysis with R (R Core Team, Vienna, Austria) and clusterProfiler package [23–25]. All database, raw MS files, and search result files in this study have been uploaded to PRIDE (PXD029143).

### Statistical analysis

All data analyses were performed using *R* and code was deposited in GitHub(https://github.com/AyassBioscience/plos_proteomic_nasal_R). Proteins between samples were classified as significantly changed if log_2_ FC higher than 1.3 and *p* value less than 0.05. Epidemiological analyses were conducted to identify proteins that showed a statistical difference between gender and age groups using the non-parametric statistical analysis (e.g. Mann-Whitney U test and Kruskall-Wallis test following Dunn ‘s post hoc test) performed with SPSS for windows (Version 23 inc., Chicago, IL, USA).

## Results

### Characteristics of patients

**Fig 1:**
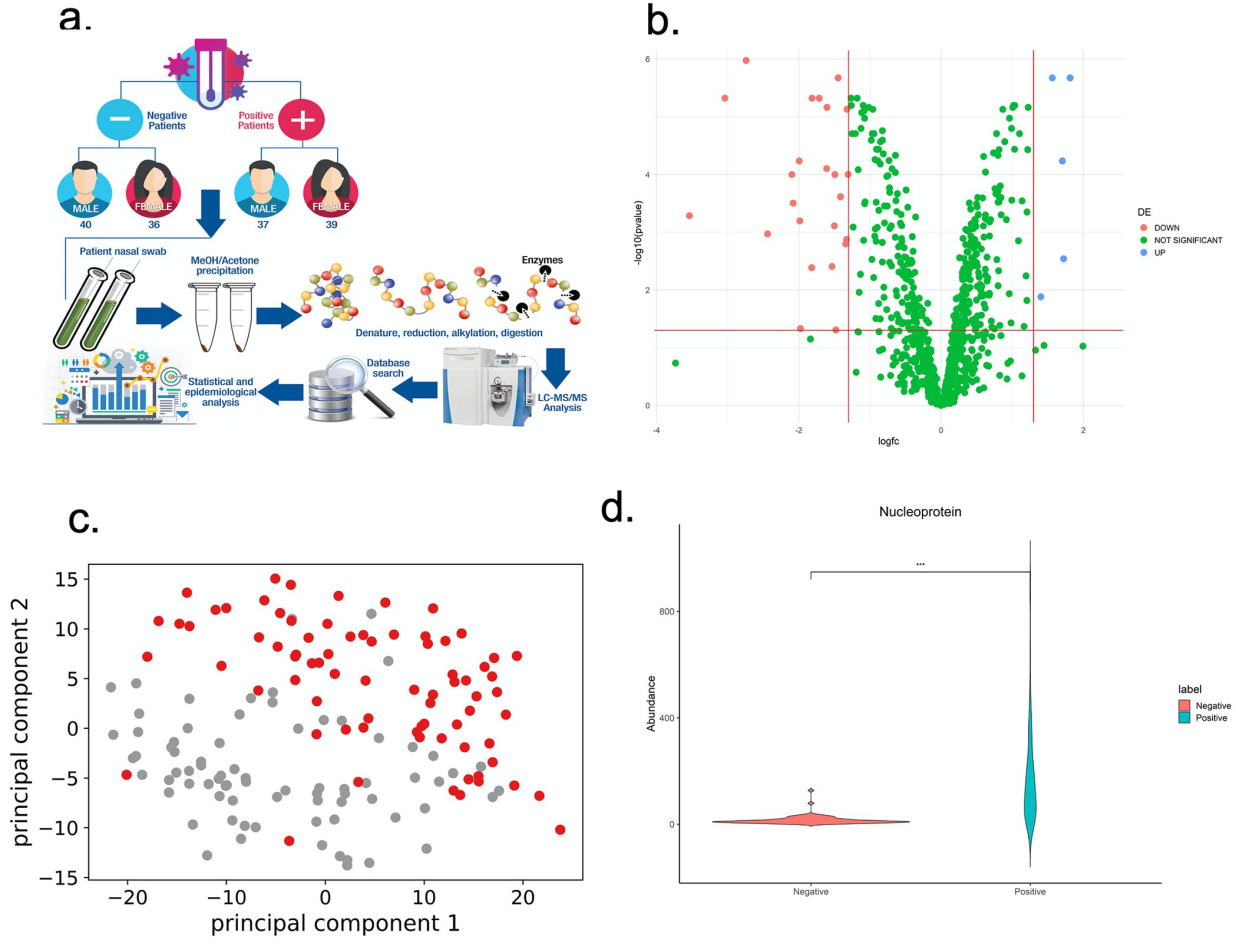
a. Overview of the workflow for data collection and proteomics analysis process. b. Volcano plot of mass spectrometry identified proteins in human nasopharyngeal environment. Proteins that were significantly up-regulated (log_2_ FC > 1.3, *p* < 0.05) in SARS-CoV-2 positive patients were highlighted in blue, and proteins that were significantly down-regulated log_2_ FC < −1.3, *p* < 0.05) were highlighted in red. c. Principal Component Analysis indicated SARS-CoV-2 positive patients (red) clustered differently from negative patients (grey). d. Nucleoprotein was identified in SARS-CoV-2 samples and low confidence peptide background resulted from algorithm was identified in negative samples.

76 patients were included in both negative and positive groups, and all samples were processed in duplicate as shown in Fig 1. Among the positive group, 51% of patients were female, while 49% were male. Two RT-PCR primers (N1,N2) from two different regions of SARS-CoV-2 virus RNA were used for detection of SARS-CoV-2 in patient samples. The median CT value was 13.15 (N1) and 14.57 (N2) (S1 Table in S1 File). Patients’ symptoms ranged from mild to severe, and included fever, chest pain, shortness of breath, cough, body pain, loss of smell and taste, headache, fatigue, etc.

### Up-regulated proteins from the nasopharyngeal proteome in COVID-19 patients

In order to maximize the amount of information obtained from the proteomics study and to minimize the loss of valuable information, data analysis was performed in two different ways: by identifying biomarkers of COVID-19 that are detected in positive samples only and by identifying biomarkers that are significantly up- or down- regulated as compared to the negative samples. For the first approach, proteins that were identified only in positive patients group were studied by setting a criteria of identifying proteins that were missed in 90% of negative samples and detected in 90% of positive samples. A total of 23 proteins were identified using this criteria as indicated in S2 Table of S1 File. A literature search of the relationship between these identified proteins and SARS-CoV-2 was conducted. Among the proteins, thimet oligopeptidase, an oligopeptidase that can convert angiotensin I into angiotensin 1–7, was only identified in SARS-CoV-2 positive samples, which is in consistent with the evidence that COVID patients have significantly increased circulating levels of angiotensin 1–7 and decreased angiotensin II [26]. Furthermore, inhibitors of Calpain-13, a calcium-activated neutral proteinase, and Kallikrein-14, a serine-type endopeptidase with a dual trypsin-like and chymotrypsin-like substrate specificity, have been investigated as a treatment for COVID-19 disease [27, 28]. Not all of the proteins in S2 Table in S1 File have been identified as COVID-19 biomarkers at this time but it is reasonable to believe that a more thorough investigation may them to be biomarkers and targets for treatment of COVID-19 disease (S2 Table in S1 File).

Criteria for the second approach consisted of a label-free quantification to identify proteins with a significant fold change (*p* < 0.05, log_2_ FC > 1.3) in SARS-CoV-2 positive patient samples compared to the negative. A total of 519 proteins from the analysis of 152 nasopharyngeal samples were identified with high confidence (FDR < 0.01) and with at least two peptides per protein (S3 Table in S1 File, Fig 1b). All database, raw MS files, and search result files in this study were uploaded to PRIDE (PXD029143). SARS-CoV-2 positive samples clustered differently from negative samples using hierarchical cluster analysis of the differentially expressed proteins (Fig 1c). A varied amount of nucleoprotein from SARS-CoV-2 was identified in all positive patient samples compared to negative patient samples, which only showed background signal (Fig 1d). Published highly expressed signature nasopharyngeal proteins [18], including upper track epithelial marker (BPI fold containing family A member 1, mucin 5, Keratin 13, polymeric immunoglobulin receptor, etc.), acute phase proteins (haptoglobin, complement component 3, alpha-2-macroglobulin, etc.), and phagocyte secretome (lactotransferrin, myeloperoxidase, lipocalin-1, etc.), were consistently identified across positive and negative patient samples (S4 Table in S1 File, S1 Fig).

**Fig 2:**
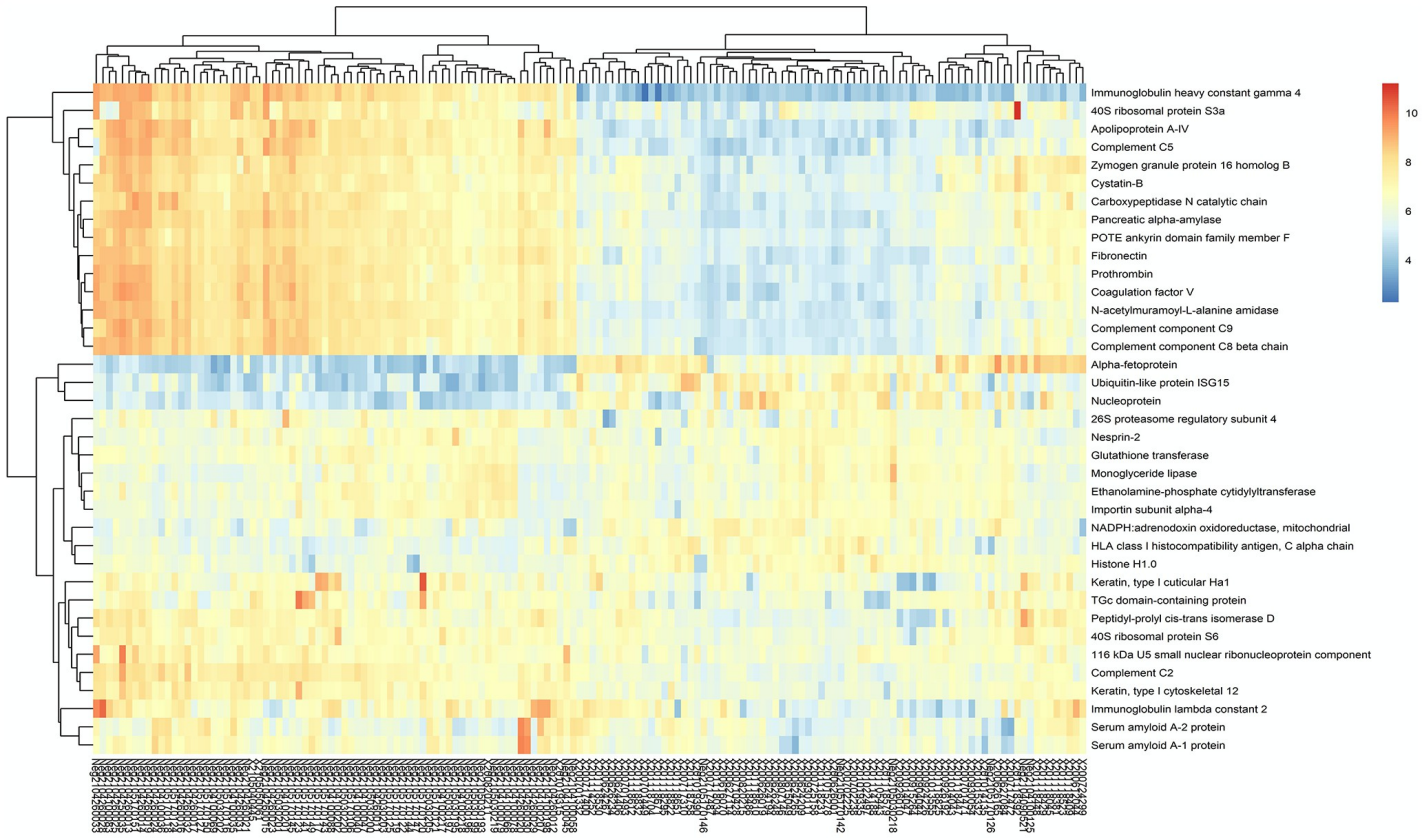
Heat map of the significantly up- and down- regulated proteins in SARS-CoV-2 positive patient samples. Different colors indicated the KNN imputed and VSN normalized abundance of proteins in different patient samples.

Using the reproducibility-optimized test statistic (ROTS), 6 proteins were found significantly up-regulated (*p* < 0.05, log_2_FC>1.3) and 31 proteins were significantly down-regulated (*p* < 0.05, log_2_FC <−1.3) in SARS-CoV-2 positive patient samples as compared to negative (Fig 2). The detailed protein list can be found in S5 Table in S1 File. Alpha-fetoprotein (AFP), a commonly used hepatocellular carcinoma biomarker [29], is the most upregulated protein identified in COVID-19 positive patient nasopharyngeal samples (*p* < 0.0001, log_2_FC = 2.26). The elevation of most serum cancer biomarkers correlates with severity of COVID-19 [11] except AFP. However, AFP in nasal environment was indeed significantly upregulated as shown in this study. High levels of AFP are also indicative of a patient with chronic liver disease and patients with such an existing condition usually have bad prognosis for COVID-19 as well [30].

An increase in ISG15 in the nasopharyngeal in SARS-CoV-2 positive patients was observed in this study, which is consistent with the increased ISG15 in the serum of patients with COVID-19 in other studies [31, 32]. Meanwhile, other research have indicated that ISG15 plays a key role in mediating and regulating host response to viral infection. SARS-CoV-2 induces human macrophages to secrete ISG15 preferentially and act as a cytokine in amplification of SARS-CoV-2 induced inflammation [31].

HLA class I histocompatibility antigen molecules are present on all nucleated cells and their increased levels can suggest an active immune response [33]. HLA molecules play an important role in the immune response by presenting endogenous peptides to cytotoxic CD8+ T cell. The HLA and related polymorphisms can influence susceptibility, progression and severity of SARS-CoV-2 infection as shown by multiple studies. Patients with mild disease present Class I HLA molecules with a higher theoretical capacity for binding SARS-Cov-2 peptides and showed greater heterozygosity when comparing them with moderate and severe groups [34]. Other studies also suggested increased risk of severe clinical course of COVID-19 in carriers of HLA-C*04:01 [35, 36].

The degradation product of Histone H1, free Histone H1.0, was found significantly upregulated in SARS-CoV-2 positive patient in the nasal environment in this study as well. Virus was known to incorporate their own genome into human genome and affect the epigenetic modifications that can affect the human genome, which includes DNA methylation, histone modifications (e.g., acetylation, citrullination and phosphorylation) and nucleosome remodeling [37]. SARS-CoV-2 disrupt host cell epigenetic regulation (e.g. DNA methylation and histone modification) and affect immune response as summarized in an review [37]. Specifically, Histone H3 was significantly increased in serum samples of SARS-CoV-2 positive ICU patients than the control samples [38]. Also, increased circulating histone levels in serum were found positively correlated with the severity of COVID-19 and the extent of coagulation activation and inflammation [39].

The last but not least upregulated protein in this study is NADPH:adrenodoxin oxidoreductase, which is the first electron transfer protein in all the mitochondrial P450 systems including cholesterol side chain cleavage in all steroidogenic tissues [40]. The relationship between COVID-19 and this protein has not been studied yet.

### Down-regulated proteins from the nasopharyngeal proteome in COVID-19 patients

31 proteins (Fig 2; S5 Table in S1 File) had a significantly (*p* < 0.05) decreased expression in COVID-19 patients with a fold change higher than 1.3 [41]. Among those proteins, five of them have not been related to COVID-19 parthenogenesis, while all others were correlated to the severity of COVID-19, covering the complement and coagualation cascade, immune system, and protein lipid digestive pathway, etc.. Notably, seven are involved in the complement and coagulation cascade.

In the coagulation cascade, prothrombin was found significantly decreased in SARS-CoV-2 positive patient nasal environment in this study. An increased D-dimer level and Prothrombin Time in serum are observed as significant indicators of sever COVID-19 and poor prognosis [42, 43]. Besides, the activity of prothrombin was also elevated in COVID-19 patients with an increased amount of prothrombin fragment 1.2 in serum, which can be used as a marker identifying thrombotic manifestations in hospitalized patients with COVID-19 [44]. Coagulation factor V was showed significantly decreased in the nasopharyngeal environment, which is suggesting the an hyper-coagulation of the body. Also, the activity of factor V was shown elevated in severe COVID-19 patients, which is associated with venous thromboembolism [45]. The full range of coagulation parameters alterations in severe COVID-19 patients were also investigated by Adam et al. [46], while coagulation factor V were normal or elevated a little in four severe COVID-19 patients’ serum. What’s more, a recent study suggested coagulation factor V as an immune inhibitor that is expressed at increased levels in leukocytes of patients with severe COVID-19 [47].

Fibronectin (log_2_ FC = −1.9, *p* < 0.0001) was identified as one of the most down-regulated protein. Fibronectin is a glycoprotein that binds to membrane-spanning receptor proteins (e.g. integrins). A recent study suggested that SARS-CoV-2 spike protein showed consensus function with proteins involved in the coagulation process (e.g. fibronectin), and that therefore the SARS-CoV-2 spike protein can directly mediate protein-protein aggregation for clots formation [48], which may thereofore result in a decrease in fibronectin level. Also, as an extracellular matrix protein, fibronectin can excessively deposit in lung and indicate a pulmonary fibrosis, which incident rate at discharge was high in severe COVID-19 cases [49, 50]. Here in our study, a significantly decreased fibronectin was find in the upper respiratory tract of SARS-CoV-2 positive patient and may suggest a pulmonary fibrosis risk at later stage of this patient. A recent clinical study suggested that the inhalation of fibronectin is effective for the treatment of COVID-19, where manifestations of patients showed a dyspnea improvement, cough reduction, sputum reduction, pulmonary exudate reduction, sputum smooth and a decreased CT imaging lesion [51].

The complement system is an essential component of the innate immune system. The three complement pathways (classical, lectin, alternative) are directly or indirectly activated by the SARS-CoV-2 as summarized elsewhere [52]. In severe COVID-19 patietns, overactivation of the complement system may contribute to the cytokine storm, endothelial inflammation (endotheliitis) and thrombosis [52]. Among all factors, the classical and lectin pathways converge into the cleavage of Complement C2 and C4 [53], and therefore, the significantly decreased C2 in this study clearly indicated an activation of the complement cascade. One study showed both C3 and C4 concentrations were significantly lower in patients with high disease severity or non-survivor status than patients with low severity or survivor status [54]. However, another study stated the levels of C3 and C4 were increased in 57.2% and 36.9% of COVID-19 patients comparing to the negative controls [55]. In the meantime, de Latour and others also found the the systemic C5 cleavage during SARS-CoV-2 infection as indicated by the increased level of circulating sC5b-9 in 64% of the patients, which is in consistent with our significantly decreased C5 in nasopharyngeal environment. As a results, some studies suggested blocking complement factor C5 with different blockers seems to be a promising method to treat COVID-19 severe patients [55–57]. Complement C6, C7, C8 and C9 together with C5a and C5b forming the membrane attack complex (MAC) C5b-9 in serum, but another study indicated there is a general upregulation of complement system proteins, including MAC proteins such as C5, C6, and C8 [58].

For the immune system, we interestingly we found that IgG4 decreased more than 3.5-fold (*p* < 0.0001) in SARS-CoV-2 positive patient nasopharyngeal environment. IgG4 antibody, a membrane-bound and secreted glycoprotein produced by B lymphocytes, mediates the activation of complement activation as well as a wide variety of diseases [59]. We suspect that antibodies increase upon viral infection, but IgG4 showed a significant decrease in the positive patient samples with convincing data quality from mass spectrometry data (S2 Fig). In serum, IgG4 is not detectable in COVID-19 patients by using Enzyme-linked Immunosorbent Assay or microsphere immunoassay [60, 61], while other study showed a decreased IgG4 in serum in ICU COVID-19 patients [62]. Another study indicated COVID-19 patients who died during 8–14 and 15–21 days also showed higher anti-RBD IgG4 levels in comparison with the recovered, suggesting that some life-threatening patients can elicit IgG4 to RBD response in the first weeks of symptoms onset [63]. A couple of studies have found that patients with IgG4-related disease, an immune-mediated multi-organ, chronic and progressive disease, are more susceptible to SARS-CoV-2 infection [64]. Masset group recently reported a case of relapse of IgG4 related nephritis following mRNA COVID-19 vaccine administration, which also highlighted the possible link between SARS-CoV-2 infection and IgG4 related disease [65]. More in-depth biological study is needed to investigate whether COVID-19 has special mechanisms to inhibit the normal production of IgG4. To further investigate the relationship between mucosal immune response and COVID-19 disease, information related to immunoglobulin heavy constant chains was extracted and summarized in S3 Fig. We found that IgG1, IgG2, IgG3, and IgM levels increased in positive patient samples, while IgA showed a slight increase without statistical significance between the positive and negative groups (S3 Fig). This supported the fact that the nasal mucosal immune system plays a critical role in virus clearance and may explain why some COVID-19 patients present with no symptom at all [14].

### Pathways enriched in COVID-19 patients

**Fig 3:**
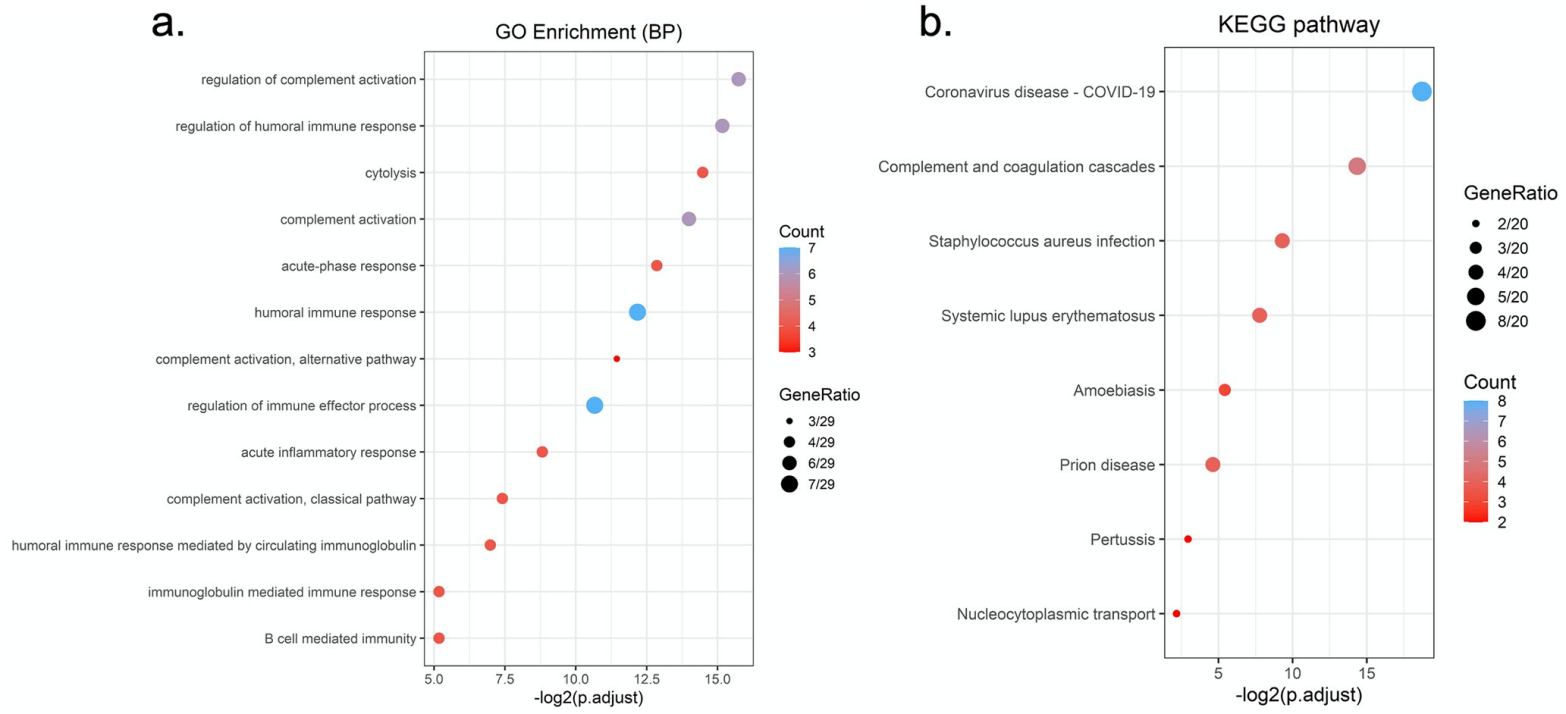
Proteomics alterations, associated pathways and diseases. (a) Shows the top 13 Pathways enriched in significantly up- and down- regulated proteins using Gene Ontology (GO) biological process (BP) Enrichment. GO-based enrichment analysis of DEPs shown in the term of biological processes (two-sided hypergeometric test; *p* < 0.05) and the number of counts (Count >3). GO terms were sorted by adjusted p-values using the Benjamini-Hochberg method. (b) Shows the top 8 diseases associated with the significantly up- and down- regulated proteins using the KEGG pathway. KEGG-based enrichment analysis of DEPs (two-sided hypergeometric test; *p* < 0.05) and the number of counts (Count > 2). KEGG terms were sorted by adjusted p-values using the Benjamini-Hochberg method.

**Fig 4:**
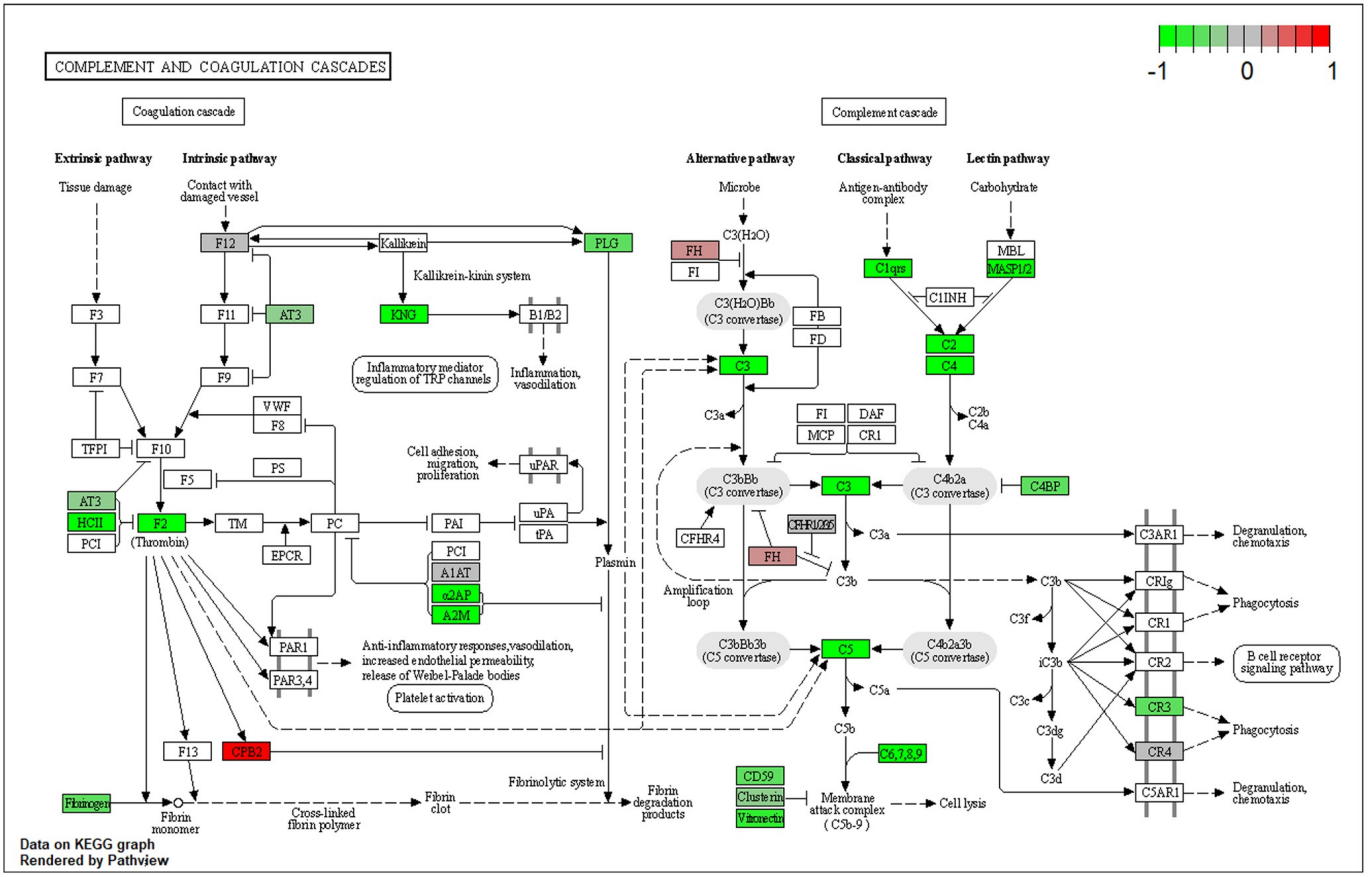
Proteins detected in this study that related to complement and coagulation cascade. Up-regulated proteins are highlighted in red and down-regulated proteins are highlighted in green.

Proteins identified in this study were subjected to KEGG and GO enrichment analysis. The complement and coagulation cascades were enriched in both pathway analyses and had the second highest enrichment ratio scores in KEGG pathway analyses (5/20) next to SARS-CoV-2 disease (Fig 3). A total of seven proteins involved in complement and coagulation cascade were identified across positive and negative samples, where four complements (C2, C5, C8, C9) and three coagulation-related factors (factor V, fibronectin, prothrombin) were identified with significantly changed ratios between positive and negative patient samples (S5 Table in S1 File, Fig 4). Among all proteins that were detected in this pathway, the decreased abundance of the substrate and the increased abundance of the end products suggested an activation of the complement and coagulation cascade, which is consistent with published studies and clinical data of COVID-19 patients with coagulation problems, such a prolonged prothrombin time (PT) and increased fibrin degradation products [9, 43, 66, 67]. Serum proteomics and clinical data studies found that complement and coagulation activation play a big role in COVID-19 pathogenesis and coagulopathy has been described in up to 50% of severe COVID-19 [10, 46]. The transcriptomic profiling of COVID-19 patients also suggests activation of complement and coagulation cascades in addition to globally dysregulated immune pathways [68].

Complement activation, humoral immune response, cytolysis, and acute-phase response were identified as the most altered pathways upon SARS-CoV-2 viral infection in the nasopharyngeal environment and they were related to each other via important genes as shown in S4 Fig. Complement factors C5, C8b, and C9 linked complement activation, cytolysis, and humoral immune response together. Complement C2 and Carboxypeptidase N submit 1 linked immune response to complement activation. Acute-phase response was connected to all other pathways through coagulation factor 2.

### Outcome in COVID-19 patients

**Fig 5:**
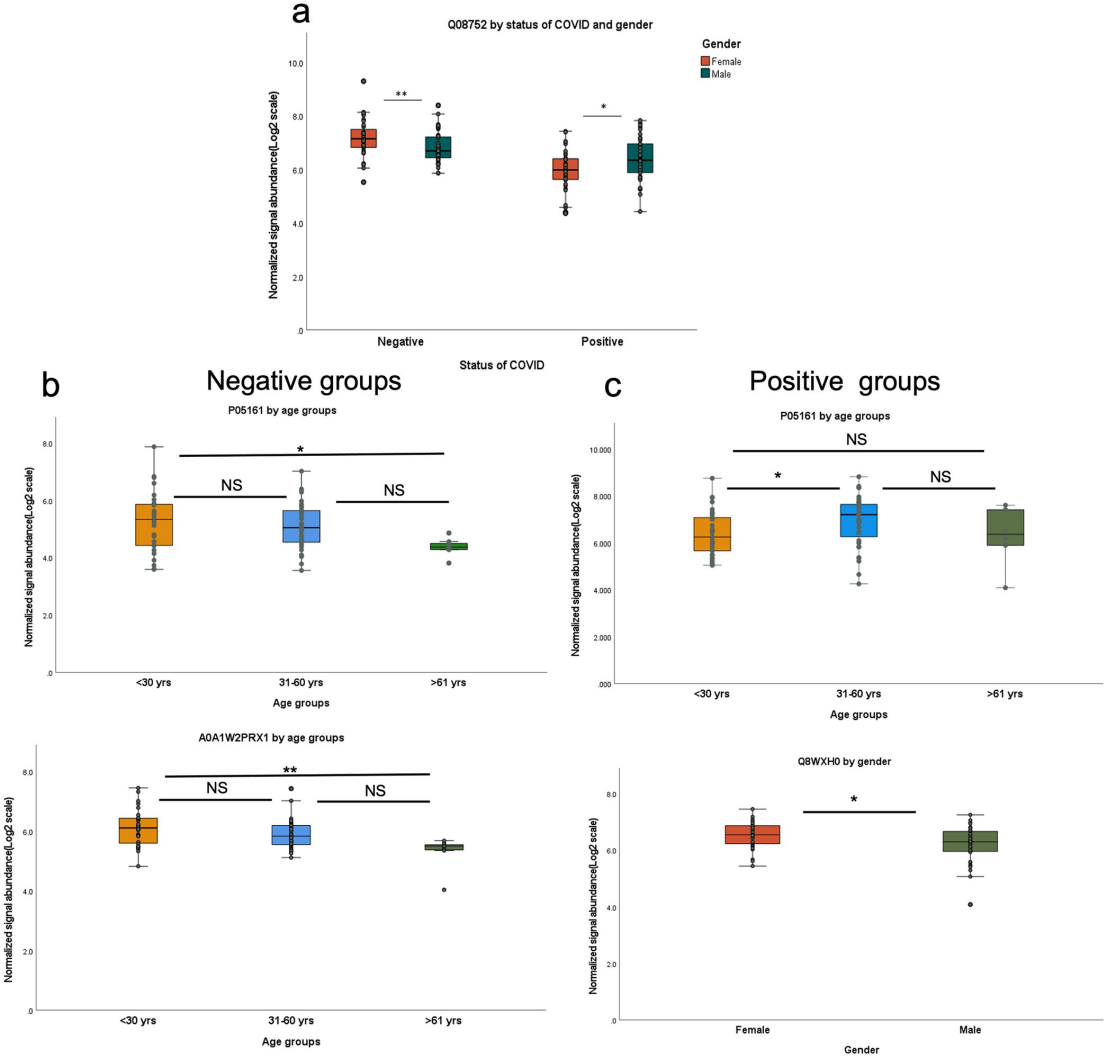
Box plots of proteomics by gender and age. a) Box plot represents significant difference of Q08752 (PPlase D) by gender in both SARS-CoV-2 negative groups(*U* = 467, *p* = 0.008) and positive groups(*U* = 504, *p* = 0.024). b) Box plot represents significant difference of proteins in the negative groups. The difference was indicated in P05161 (Ubiquitin-like protein ISG15)(*χ*^2^(2) = 6.029, *p* = 0.049) and A0A1W2PRX1 (HLA class I histocompatibility antigen) (*χ*^2^(2) = 10.584, *p* = 0.005) by age groups. C) Box plot represents significant difference of proteins in the positive groups. The difference was indicated in P05161 by age groups (*χ*^2^(2) = 5.998, *p* = 0.05) and Q8WXHO (nesprin-2) by gender (*U* = 488, *p* = 0.015). The Mann-Whitney test was conducted to compare the difference of proteins between gender. Krustal-Wallis H test following Dunn’ s post-hoc test was conducted to compare the difference of proteins between age groups. *denotes *p* ≤ 0.05 and **denotes *p* < 0.01.

As part of this study, all 37 proteins that showed significant up- or down- regulation were compared between gender and age groups by using non-parametric statistics tests. A Mann-Whitney test indicated that PPIase D protein (Uniprot ID: Q08752) was significantly higher in females than males in the negative groups (*U* = 467, *p* = 0.008); however, it was significantly lower in females than in males in the SARS-CoV-2 positive groups (*U* = 504, *p* = 0.024)(Fig 5a). Yamamotoya et al. study demonstrated that Pin 1, one of the family of peptidyl-prolyl isomerase (PPlases), enhanced the proliferation of SARS-CoV-2 by increasing production of inflammatory proteins in the host cells, and Pin 1 inhibition may be a promising therapy against COVID-19 [69]. Our result showed a flip-over change of PPlase D in males before and after SARS-CoV-2 viral infection, which may provide a possible clue as to why men experience more severe COVID-19 outcomes than women [70].

A Krustal-Wallis H test provided significant evidence of a difference in Ubiquitin-like protein ISG15 (Uniprot ID: P05161) between the mean ranks of at least one pair of age groups (<30 years, 31–60 years, >61 years) in both SARS-CoV-2 negative groups (*χ*^2^(2) = 6.029, *p* = 0.049) and positive groups (*χ*^2^(2) = 5.998, *p* = 0.05). Dunn’s pairwise test was carried out for three pairs of groups. Dunn’s test indicated that Ubiquitin-like protein ISG15 is significantly higher in the <30 years group than the >60 group in the SARS-CoV-2 negative groups(*p* = 0.047). Dunn’s pairwise test also indicated that Ubiquitin-like protein ISG15 is significantly lower in the <30 years group than the 30–60 years group in the SARS-CoV-2 positive groups(*p* = 0.05)(Fig 5b and 5c). There is no evidence of a difference between the other pairs. As shown in previous study, ISG15 plays a key role in mediating and regulating host response to viral infection [31, 32]. However, the relationship between age and ISG15 is uncertain; further research is needed.

At the same time, a Krustal-Wallis H test provided very strong evidence of a difference (*χ*^2^(2) = 10.584, *p* = 0.005) of HLA class I histocompatibility antigen between the mean ranks of at least one pair of age groups as well. Dunn’s pairwise test was carried out for three pairs of groups. Dunn’s pairwise test indicated that HLA class I histocompatibility antigen is significantly higher in the <30 years group than the >60 years group (*p* = 0.004)(Fig 5b). There is no evidence of a difference between the other pairs. HLA genes are recognized to be important factors in the host response to foreign pathogens. Langton’s study found that HLA influence the severity of COVID-19 infection and HLA alleles interact with age, sex and BMI to determine clinical outcomes following COVID-19 exposure [36].

A Mann-Whitney test also indicated that nesprin-2 was significantly higher in females than male (*U* = 488, *p* = 0.015) in the SARS-CoV-2 positive groups(Fig 5c). Nesprin-2 is highly expressed in skeletal and cardiac muscles and the mutation of Nesprin-2 can cause dilated cardiomyopathy [71]. A COVID cohort study by Shi demonstrated that 28% of patients exhibited myocardial injury and myocardial injury is significant associated with fatal outcome of COVID-19 [72]. However, more research is needed to clarify how gender impacts Nesprin-2 in COVID-19.

## Discussion

To date, the COVID-19 plasma/serum proteome has been investigated using various protein assays based on LC/MS methods that lead to identification of important protein biomarkers in patients with COVID-19 disease [9, 73, 74]. Our study utilized a non-invasive method of collecting samples from the nasal swabs of COVID-19 patients and allowed us to present the status of proteomics in the nasal microenvironment at the time of diagnosis of COVID-19 (acute infection phase). A study by Ting et al. revealed that the coagulation pathway plays an important role in the development of COVID outcome through the plasma proteomic pathway analysis [9]. Our nasal study showed similar findings as Ting et al. without the need to collect blood from patients and extending the exposure of healthcare workers. Also, studies have shown that there was a strikingly different cytokines profile in the nasopharynx compartment when comparing the whole-body system and the mucosal immune response to COVID-19. Studies have suggested that nasopharyngeal cytokine responses are regulated independently, and the nasopharyngeal microbiome regulates local and systemic immunity that determines the clinical outcome of COVID-19 [75–77]. Thus, proteomic analysis of nasal mucus is able to provide an in-depth insight into the upstream inflammatory process that leads to the downstream clinical manifestation of COVID-19. The study of nasal proteomics could also determine the molecular signature of importance for disease progression and to understand the molecular mechanism of the immune-related response of the SARS-CoV-2 virus.

Compared to similar studies that had a smaller sample size (9–20 samples/ group) [6, 78, 79], our study included 76 positive and 76 negative COVID-19 samples in each group. The sample size allowed us to perform proper statistical analysis and present statistically significant differences in the proteome expression between patients diagnosed with COVID-19 disease as compared to those who were not.

Although nasopharyngeal swab samples can be collected several times over the course of treatment for almost all patients, they are not favored and studied by researchers due to the variation in each sample collection. Among the challenges, the 2% FBS present in the viral transport media may mask the results of the real human nasopharyngeal proteomics; another one is the varied and limited number of human cells found in each sample which presents a huge challenge in data normlization; a third challenge is that academic research labs typically lack access to large number of patient samples while clinical labs have no proteomics profiling capabilities.

Our study overcame these challenges by employing a strict statistical criterion during data analysis stage. Data from this study mainly belong to the human proteome with minimal FBS proteome interference. We hypothesized that the mass spectrometer should be able to pick up the minor amount of human proteome in FBS background without needing to deplete serum albumin and therefore, we conduct a direct methanol/acetone precipitation of the total proteins. Our results showed high abundance proteins were mostly nasal mucosal cells markers as well as nasal secreted proteins (S3 Table in S1 File). Our sample choice has the advantage of using nasopharyngel swabs over serum/plasma samples that contain 50–60% albumin, which is known to impose a challenge in identifying low abundant proteins in LC-MS/MS studies [80]. Although it is difficult to find the ideal method to normalize the data due to the inherent heterogenicity of samples, most significantly changed marker proteins can be discovered by using a stringent statistical criteria in this study. A standard proteomics workflow entails spiking either heavy or standard peptides as an internal standard to ensure that all analyses start with the same amount of total protein. It was not possible to do that in this study since it is hard to control the percentage of human cells in the nasopharyngeal swab samples. Instead, a global normalization to the total peptide count (TPC) was used to adjust injection to injection variations, and the ROTS package was used to further normalize the data and only identify the most significant changed proteome in samples with some inconsistent small anti-viral peptides and d-dimers sacrifised.

In this study, patient nasopharyngeal swabs used for RT-PCR test were re-utilized for a bottom-up proteomics test. A series of biomarkers were identified from positive nasopharyngeal environment compared to the negatives. A lot of evidence was presented to explain how SARS-CoV-2 may trigger activation of complement and coagulation cascades [10]. According to our study, complement and coagulation cascades activation happens as early as the point of entry, and treatment related to this pathway may be given at this point to prevent later complications and disease progression. Together with a comprehensive set of patient clinical history data, those biomarkers could be used to predict patient prognosis as soon as the virus is detected in the nasopharyngeal environment, as well as prevent mortality and complications that might happen later.

The mucosal immune system has largely been neglected, but research has revealed its valuable role in combating SARS-CoV-2 [14]. We found that IgG4 (log_2_ FC = −3.5, *p* < 0.0001) is significantly decreased in the nasopharyngeal environment upon SARS-CoV-2 infection at the point of entry, which may reveal some unique changes of the mucosal immune response after SARS-CoV-2 viral contamination. The mucosal immune system is very important for fighting against viral infection and many groups are working hard to develop nasal spray vaccines to better fight against the fast mutations in SARS-CoV-2. It would be great to clearly reveal how the mucosal immune system changes upon SARS-CoV-2 infection since the mucosal immune system plays such an important role in viral clearance and may explain why symptoms vary so much between patients. However, there is a great deal of heterogenicity in the human mucosal immune systems due to age, gender, and health conditions (e.g. coinfection or other underlying conditions). All of these conditions affect the number of antibodies in the upper-airway and add an extra layer of difficulty to the analysis of the nasopharyngeal proteomics data. Therefore, we can hardly tell how other important antibodies change upon viral infections with statistically significant values (e.g. IgA showed a slightly increased trend but it is hard to confirm due to the inherent sample variations).

One potential limitation of the study is that the positive subjects selected had a CT value of 15 or less. However, this study is an observational one conducted at the early stages of the COVID-19 pandemic when almost all symptomatic patients had presented COVID-19 disease. The patients with high viral load (low CT value) were chosen due to their potentially higher risk of developing and achieving severe clinical outcomes. Thus, this study selected and presented only the results of cases with CT value less than 15 for patients that were in the acute phase of infection and is lacking information related to the proteomic profile difference between patients with a low viral load (higher CT value) and COVID-19 negative patients. This study also compared the proteomics difference between COVID-19 positive and COVID-19 negative samples, and the pathway analysis showed that the complement and coagulation cascades played an important role in the acute infection phase of COVID-19. However, 7 out of 13 Go enrichment pathways showed that the proteomics profile was related to inflammatory response, humoral immune response, or B cell mediated immunity, etc. (Fig 3), suggesting that these biomarkers could be found in other respiratory illnesses. With the lack of control samples represented by nasopharyngeal specimen collected from patients with other respiratory illness such as influenza A/B or respiratory syncytial virus, we cannot claim that these biomarkers are specific to COVID-19 disease. To continue to gain an in-depth understanding of the proteomics in the nasal environment of COVID-19 patients, a separate study of samples collected from patients with lower SARS-CoV-2 viral loads (higher CT values) and other inflammatory diseases/respiratory illnesses will be conducted in the future.

In summary, our study used MS-based proteomic methods and provided an unbiased, hypothesis-free investigation of the nasal mucosal proteome. Down-regulation of IgG4 correlates with a bad outcome in COVID-19 patients and the decreased level of IgG4 at the point of entry could be useful for early detection of adverse immune response in COVID-19 patients and predict their ultimate outcome. Decreased coagulation factor 5 with a decrease in fibronectin suggested an increased chance of thrombotic events in COVID-19 patients, a relative common complication seen in COVID-19 patients [81]. Other proteins successfully identified in this study could also be promising biomarkers for early diagnosis of COVID-19, disease monitoring, as well as drug targets.

## Conclusion

In conclusion, our study demonstrated that mass spectrometry-based proteomics analysis of nasopharyngeal swabs can be a powerful approach to evaluate host proteome changes due to viral infection at the point of entry and may provide valuable insights into COVID-19 pathogenesis. Down-regulation of IgG4 in nasopharyngeal environment suggested mocusal immune abnormalities upon SARS-CoV-2 viral infection at the point of entry and decreased coagulation factor 5 and fibronectin suggested an increased chance of thrombotic events in COVID-19 patients. Other proteins successfully identified in this study could also be promising biomarkers for early COVID-19 diagnosis, mucosal immune targeted medicines, vaccines, as well as prognosis predictors.

## Supporting information

S1 Fig Trend of different nasal protein markers upon SARS-CoV-2 contamination in patient nasopharyngeal swab samples. Protein list refers to S4 Table in S1 File.
(TIF)

S2 Fig MS2 fragment match spectrum of high confidence peptides from immunoglobulin heavy constant gamma 4 (A0A286YFJ8). Seven high confidence peptides with 413 PSM (peptide spectra matches) were found using QE, only four exemplar peptides are shown in this figure.
(TIF)

S3 Fig Trend of different immunoglobulin upon SARS-CoV-2 contamination in patient nasopharyngeal swab samples. a. IgA1, b. IgG1, c. IgG2, d. IgG3, e. IgG4, f. IgM.
(TIF)

S4 Fig Interaction of significantly changed genes and their linked pathways. (TIF)

S1 File (XLSX)
