# Supplementary figures and images for: Noninvasive nasopharyngeal proteomics of COVID-19 patient identify abnormalities related to complement and coagulation cascade and mucosal immune system
Source: PLoS One. 2022 Sep 12;17(9):e0274228. doi: 10.1371/journal.pone.0274228 (PMC9467311; doi:10.1371/journal.pone.0274228)

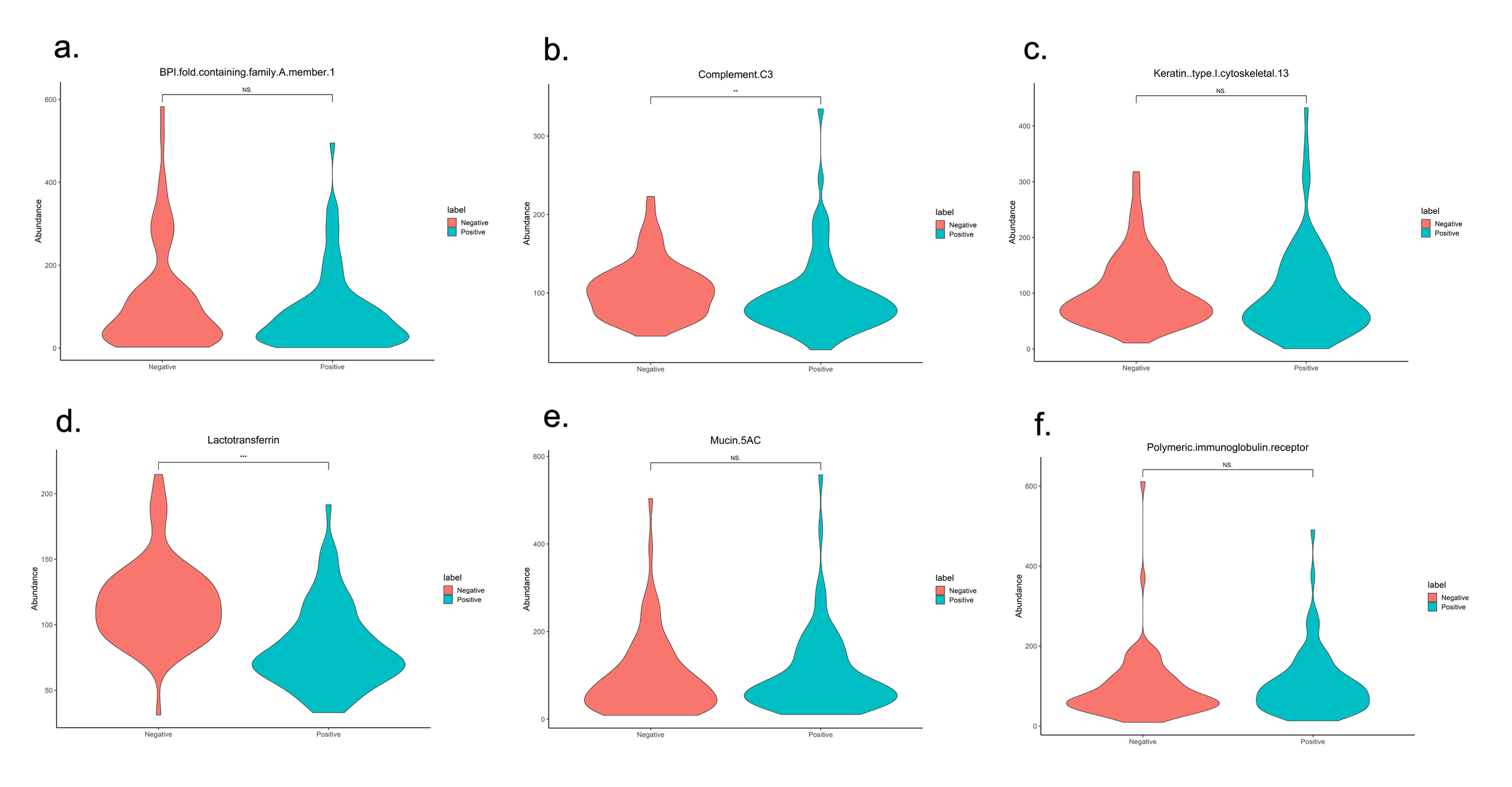

Supplement: S1 Fig — Protein list refers to S4 Table in S1 File. (TIF) [file pone.0274228.s001.tif]

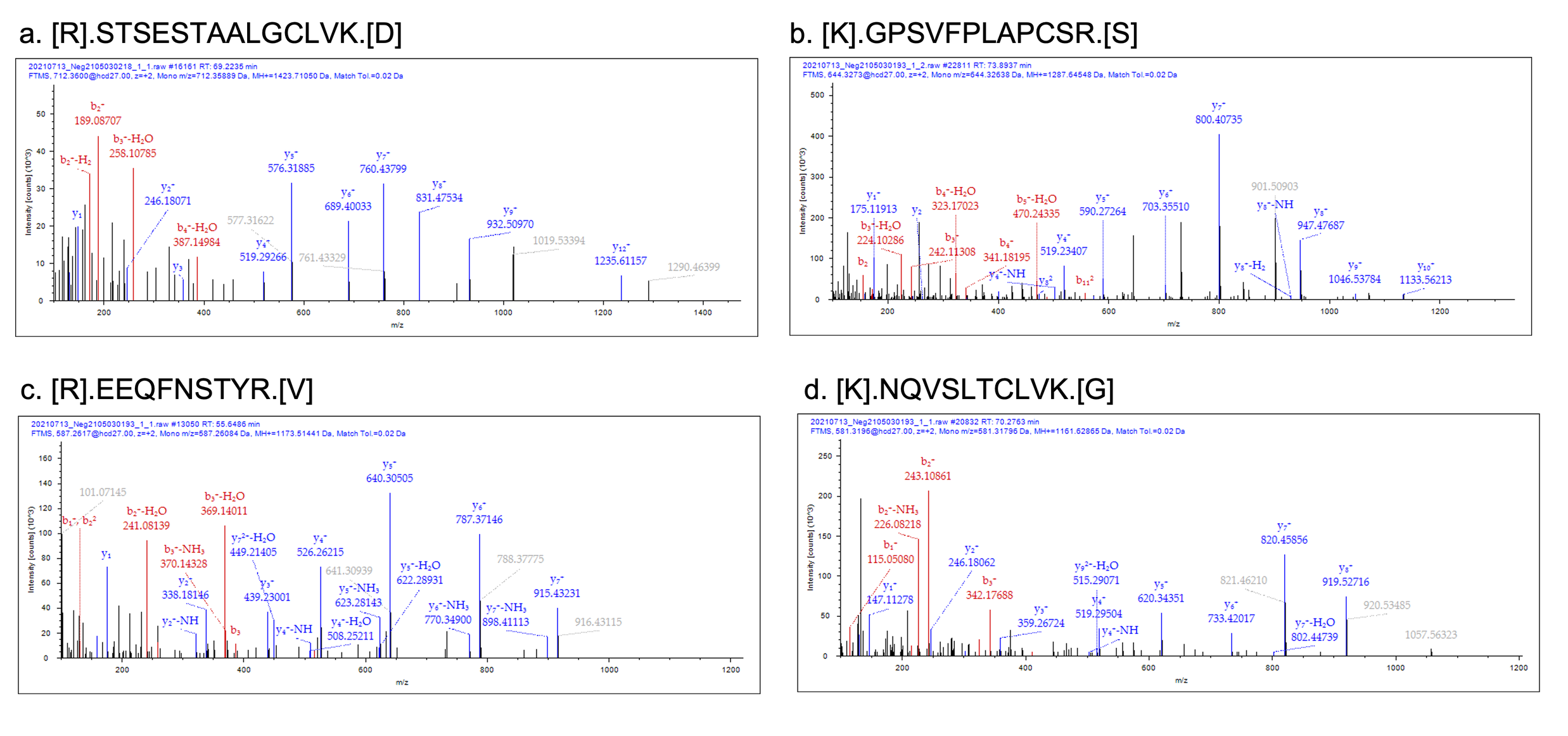

Supplement: S2 Fig — Seven high confidence peptides with 413 PSM (peptide spectra matches) were found using QE, only four exemplar peptides are shown in this figure. (TIF) [file pone.0274228.s002.tif]

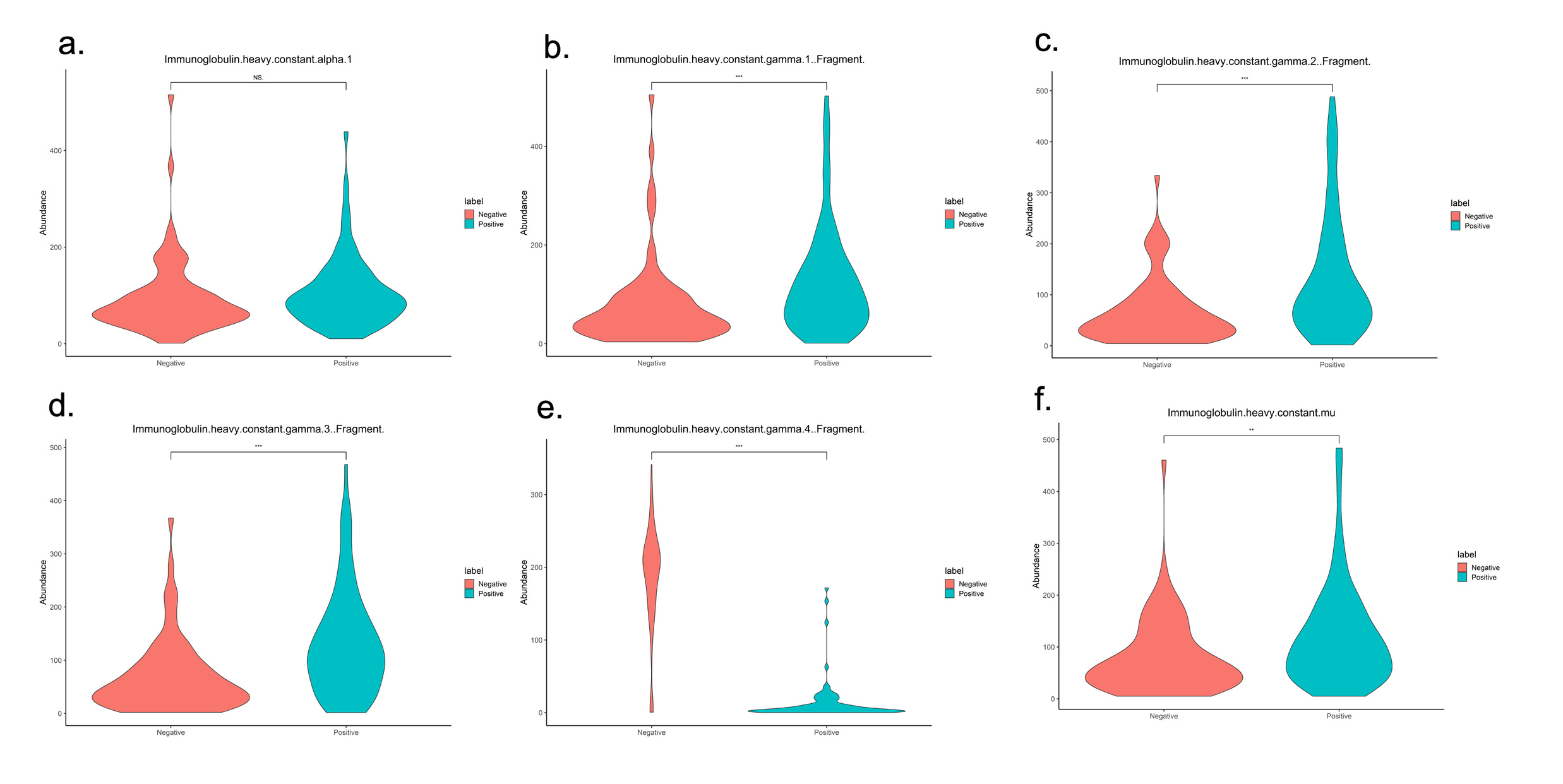

Supplement: S3 Fig — a. IgA1, b. IgG1, c. IgG2, d. IgG3, e. IgG4, f. IgM. (TIF) [file pone.0274228.s003.tif]

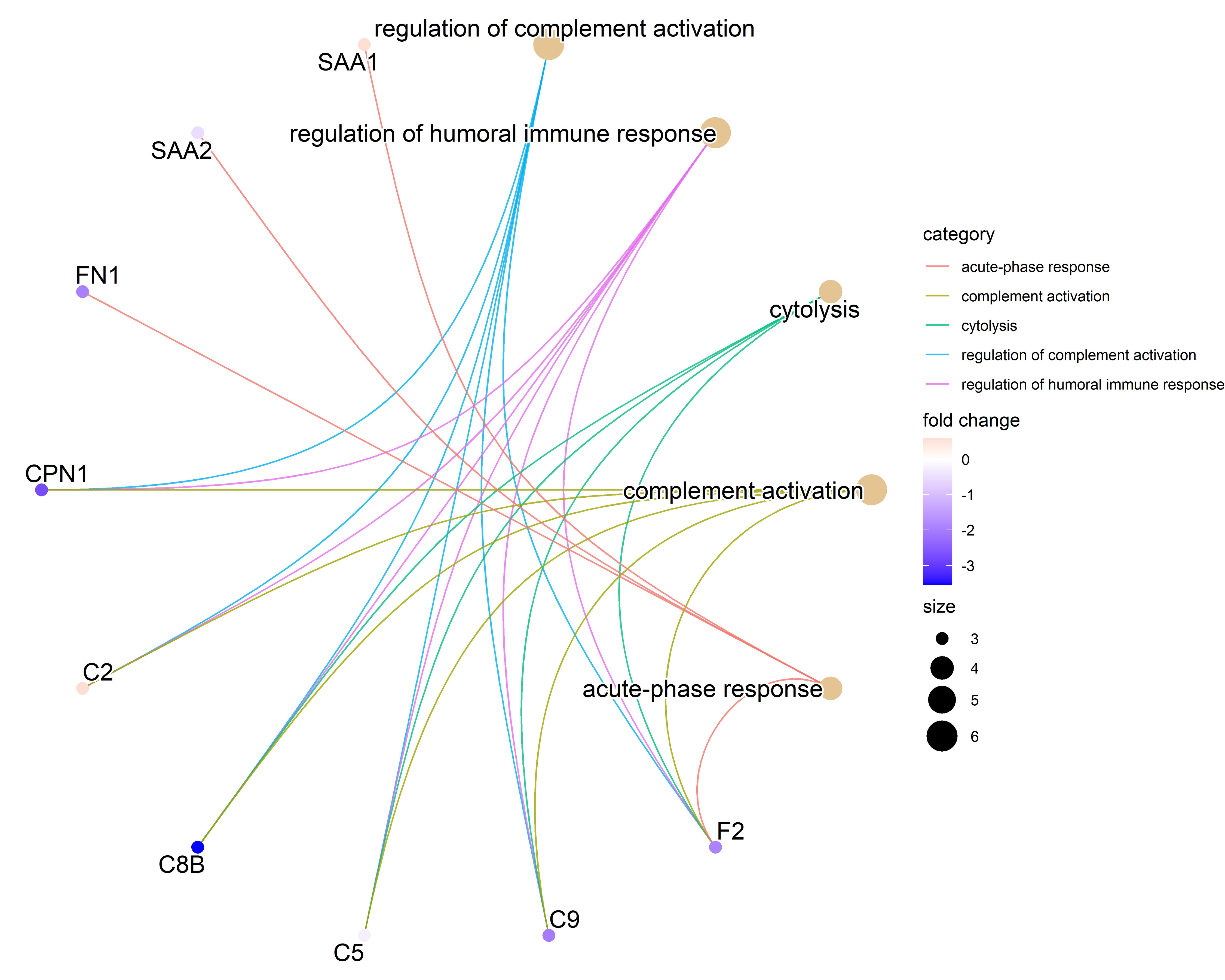

Supplement: S4 Fig — (TIF) [file pone.0274228.s004.tif]
